# Supplementary material for: A Critical View of the Application of the APEX Software (Aqueous Photochemistry of Environmentally-Occurring Xenobiotics) to Predict Photoreaction Kinetics in Surface Freshwaters
Source: Molecules. 2019 Dec 18;25(1):9. doi: 10.3390/molecules25010009 (PMC7017383; doi:10.3390/molecules25010009)
Supplement: Supplementary file 1 [file molecules-25-00009-s001.zip › 3.2.4_gcc-4.4.0.zip!/3.2.4_gcc-4.4.0/3.2.4_gcc-4.4.0/doc/octave/html/interpreter/Evaluation-in-a-Different-Context.html]

Evaluation in a Different Context - Untitled


Previous: Calling a Function by its Name,
Up: Evaluation


---

### 9.2 Evaluation in a Different Context

Before you evaluate an expression you need to substitute
the values of the variables used in the expression. These
are stored in the symbol table. Whenever the interpreter
starts a new function it saves the current symbol table
and creates a new one, initializing it with the list of
function parameters and a couple of predefined variables
such as `nargin`. Expressions inside the function use the
new symbol table.

Sometimes you want to write a function so that when you
call it, it modifies variables in your own context. This
allows you to use a pass-by-name style of function,
which is similar to using a pointer in programming languages such
as C.

Consider how you might write `save` and `load` as
m-files. For example,

```
     function create_data
       x = linspace (0, 10, 10);
       y = sin (x);
       save mydata x y
     endfunction
```

With `evalin`, you could write `save` as follows:

```
     function save (file, name1, name2)
       f = open_save_file (file);
       save_var(f, name1, evalin ("caller", name1));
       save_var(f, name2, evalin ("caller", name2));
     endfunction
```

Here, caller is the `create_data` function and `name1`
is the string `"x"`, which evaluates simply as the value of `x`.

You later want to load the values back from `mydata`
in a different context:

```
     function process_data
       load mydata
       ... do work ...
     endfunction
```

With `assignin`, you could write `load` as follows:

```
     function load (file)
       f = open_load_file (file);
       [name, val] = load_var (f);
       assignin ("caller", name, val);
       [name, val] = load_var (f);
       assignin ("caller", name, val);
     endfunction
```

Here, caller is the `process_data` function.

You can set and use variables at the command prompt
using the context base rather than caller.

These functions are rarely used in practice. One
example is the `fail (`code`,` pattern`)` function
which evaluates code in the caller's context and
checks that the error message it produces matches
the given pattern. Other examples such as `save` and `load`
are written in C++ where all Octave variables
are in the caller context and `evalin` is not needed.

— Built-in Function: **evalin** (context, try, catch)  
> Like `eval`, except that the expressions are evaluated in the
> context context, which may be either `"caller"` or
> `"base"`.

— Built-in Function: **assignin** (context, varname, value)  
> Assign value to varname in context context, which
> may be either `"base"` or `"caller"`.
